# Digital Media Used in Education: The Influence on Cyberbullying Behaviors among Youth Students

**DOI:** 10.3390/ijerph20021370

**Published:** 2023-01-12

**Authors:** Omar A. Alismaiel

**Affiliations:** College of Education, King Faisal University, Al-Ahsa 13982, Saudi Arabia; oalismaeel@kfu.edu.sa

**Keywords:** digital media, cyber harassment, stalking, bullying, as well as the ethical implications for student behavior, psychological health

## Abstract

Students, colleagues, and other members of society are increasingly using digital media. Students utilize digital media for a variety of reasons, including communication, gaming, making new friends, and simply being curious. However, there are some disadvantages to using digital media. Cyberbullying, cyberharassment, and cyberstalking are examples of useful digital media activities that can have a negative impact on digital media users and lead to societal issues. Surprisingly, limited studies have investigated cyberbullying in depth, utilizing a broad and varied sample of Middle Eastern institutions. As a result, the purpose of this study is to fill a research vacuum by questioning students’ use of digital media for cyber involvement. This research aims to create a model for assessing the ethical consequences of behaviors that directly impact students’ psychological health because of their use of digital media. The questionnaire looked at how people used digital media to engage in cyberbullying and cyber engagement, the ethical implications of bullying, and being harassed, stalked, and bullied. The study employed a quantitative questionnaire to collect data to achieve the research goal. It was given to 1012 students who are digital media users. Partial least squares (PLS) and structural equation modeling (SEM) were used to examine the data. Considering the empirical data, nearly half of the participants admitted to being harassed, stalked, or bullied on different digital platforms. The evaluation of discriminant validity is a prerequisite factor for examining possible variables’ relationships. The goodness-of-fit index indicates that the model is well-fit. Through the established model, decision-makers and school administration would be able to implement measures that would effectively reduce cyber harassment among students and improve the digital media usage experience.

## 1. Introduction

Cyberbullying is defined as the use of websites, SMS messaging, mobile phones, email, and other information and communication technologies to socially isolate, humiliate, harass, or threaten an individual [1]. When someone urges you to engage in sexual conduct via the internet or sends you unwanted, sexually explicit photographs or conversations, this is known as cyber harassment [2]. Furthermore, cyber harassment can include identity theft, online surveillance, spamming, hate speech, individual abuse, and cyberbullying. Cyber harassment affects a large number of people; in fact, in the United States alone, over 500,000 students (18+ years of age) have been recognized as victims [3]. One detrimental impact of cyber harassment, according to [3], is that people who have been harassed will often depart a social networking site (2010). Online harassment, on the other hand, has the potential to lead to suicide [4].

The similarities and distinctions between stalking and cyberstalking have recently been the subject of research [5], with issues raised concerning their similarities and differences, especially when it comes to the causes of cyberbullying rather than the expected behavioral regulation for young students [6]. Furthermore, cyberbullying has been related to poor communication among children and poor academic achievement [7]. Educators, however, are frequently blind to the impact of cyberbullying on children due to the secrecy with which it occurs [8]. In addition, many parents of teenagers are inept at monitoring or assessing their children’s internet conduct [6]. This is owing to their guardians’ frequent lack of knowledge of information and communication technology [9]. Regardless, as Shariff has shown, cyberbullying has far-reaching detrimental implications, even if they go unrecognized [10,11]. Those responsible for children must be informed of the consequences of cyberbullying to urge them to continue their education [12,13].

### Literature Review and Problem Background

Based on a longitudinal study, Reference [14] found that bullying victimization predicts ethical, behavioral, and emotional difficulties in adolescence. The majority of cyberbullying research focuses on emotional difficulties and ethical ramifications [15]. Several cyber harassment evaluations have been presented, but little effort has been taken to properly analyze the available literature to back up existing varied opinions. One point of view, for example, is whether cyber harassment is a criminal act or if it is simply a stalker empowered by technology.

Furthermore, cyberstalking is a lot more serious problem than previously considered, and it should be recognized as a major criminal issue and public health concern [16]. Cyberstalking is the term for stalking on the internet, “an extreme kind of online harassment directed at a single person, causing severe emotional distress while having no legitimate purpose. The goal is to anger, scare, and emotionally assault someone else [17]. Cyber harassment has been linked to major public health issues in previous research [18,19].

Cyber harassment may cause a wide range of psychological and emotional problems, including sadness, wrath, discomfort, anxiety, and danger for those who have been subjected to it [20,21]. Despite experiencing the same sorts or levels of victimization, it has been claimed that females and younger victims suffer more than boys and older adolescents [10,18,22]. A succession of vexing events (for example, several aggressors, anonymous harassment, and covert repetition and persistence) is likely to enhance the chance of teenage distress [19,20]. According to the study, over 15% of the people were bullied within six months, and 2.8 percent were cyberbullied [23].

According to [24], among university students, high levels of stress and depression increase the likelihood of becoming a victim of cyberbullying, while high levels of depression increase the likelihood of becoming an aggressor in cyberbullying. Compared to other life stages (childhood or adolescence) or educational cycles (primary or secondary education), the university context and the changing life phase of the students during this time have some unique characteristics that may impact cyberbullying and should be carefully taken into account. The majority of college students are currently going through an evolving stage of life known as “emerging adulthood” [25], during which new behavioral, cognitive, and emotional or affective responses are being formed in response to changing environmental demands.

Numerous studies [26,27,28] have examined the issue in youth, but recently substantial prevalence rates have also been discovered in the university setting. According to research, the prevalence of students harmed by electronic means while enrolled in higher education may range from 5 to 40% [29,30]. Using a large sample of university students from the United States in 2002, Finn [31] discovered that 10 to 15% of them reported receiving harassing or threatening emails or messages. In a sample of 420 university students, Lindsay and Krysik [32] discovered greater prevalence rates, indicating that 43.3% of the students reported having experienced cyberbullying.

Using a sample of 1925 Canadian university students, Faucher et al. [29] discovered that 24.1% of them had experienced cyberbullying in the previous year. According to Zalaquett and Chatters [30], 19% of a sample of 613 university students reported being the victims of cyberbullying, whereas 5% reported being the aggressors in cases of cyberbullying. Perhaps due to the many conceptualizations of cyberbullying, the various methodologies employed, or the calculation of the frequency required to be considered cyberbullying, the prevalence rates show quite a bit of variation.

Despite this variation, actual data support the existence of this issue at universities. Accordingly, References [33,34] show that a student’s likelihood of making a poor social and emotional adjustment to university may be predicted by their experience with cyberbullying throughout their secondary education. However, not many studies are looking into whether or not this adjustment to university life affects one’s likelihood of becoming a victim or aggressor of cyberbullying. As far as we know, the study done by [35,36] is the only one pertinent. These authors discovered that newcomer adjustment and student sentiments of well-being determine whether someone will be a victim or aggressor of cyberbullying, using a sample of 979 Brazilian and Portuguese university students.

In a survey of roughly 25,000 students, Reference [21] found that 87 percent of pupils in 25 European countries use the internet at home, while 63 percent use it at their schools. Teenagers also use a range of technologies to learn about sexual health, but digital media falls short of addressing the sexual health gap. Indeed, the characteristics of cyberbullying, stalking, and harassment as they affect students, as well as any ethical effects or behavior concerns associated with students’ adoption of digital media, require investigation from the perspective of students’ perspectives and cognizance of related variables. This may aid in developing a theoretical model that can contribute to a greater understanding of the literature.

As a result, the purpose of this research is to develop a strategy for identifying the important factors that are expected to have a major influence on reducing cyber harassment, stalking, and bullying, all of which have ethical implications for young students’ conduct. In response to identified research gaps and to stimulate future research, this study focuses on the effect of digital media use on cyberbullying behaviors among university students. There are three types of contributions: I identify the characteristics that drive cyberbullying on digital media, (ii) investigate the relationship between all of these factors, and (iii) develop a model for how digital media influences cyberbullying behavior among university students. In summary, this study aims to investigate and quantify the ethical implications of digital media use and cyberbullying behaviors that directly affect university students’ psychological health.

## 2. Research Model and Hypotheses Development

**Figure 1:**
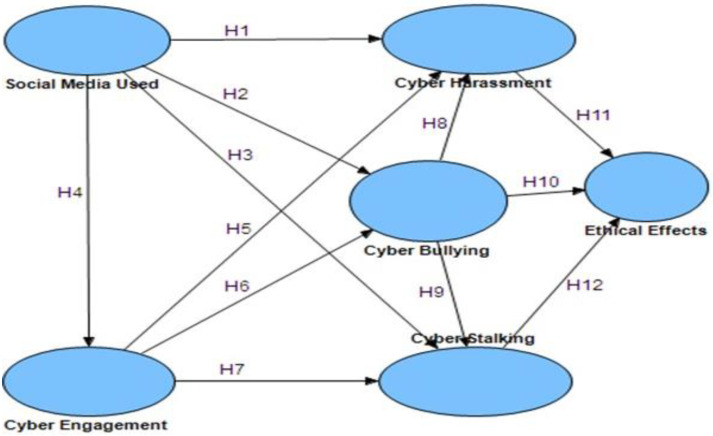
Research model.

A significant amount of diagnostic research on cyberbullying, cyber harassment, and cyberstalking is inadequate. Indeed, it appears that no study at UAE University examines the elements that impact or mitigate cyberbullying among youngsters [37]. As a result, we need to do a thorough investigation as quickly as possible to determine the factors that impact cyberbullying, cyber harassment, and cyberstalking in a university context [38]. Therefore, online participation and digital media usage are independent factors in this study, while cyber harassment, cyberbullying, and cyberstalking are mediator variables. The dependent variable is the moral effect of the action that directly impacts the students’ psychological well-being. This model will utilize the term “cyber harassment” to refer to both cyberstalking and cyberbullying. Participation in a range of digital networking organizations and online encounters with persons with dubious identities have been proven to be substantial predictors of increased sensitivity to the danger of being attacked [38]. Engaging in online behavior, also known as “cyber engagement”, is referred to as “cyber stalking” [39]. It has been recommended that schools and homes adopt an online engagement method in which children create an online agreement or contract for computer use. The contract or agreement should include specific recommendations on ethical behavior since research has shown that when children understand that bullying is against the rules and how to report it [40]. The scenario is depicted in Figure 1.

### 2.1. Digital Media Used (SM)

Users use digital media to create online communities to share information, ideas, personal messages, and other content (such as videos). Digital media is defined as “forms of electronic communications (such as websites for digital media and microblogging) through which users create online communities to share information, ideas, personal messages, and other content (such as videos)” [41]. Although there is no globally acknowledged definition of cyber harassment, it is commonly characterized as strangers or acquaintances using mobile phones, the internet, or other kinds of information and communication technology to send unwanted, insulting, intimidating, or obnoxious content [9]. Online harassment includes, as previously said, cyber harassment, identity theft, cyberstalking, spamming, hate material, individual abuses, and cyberbullying [42]. Because English is the most used language on most digital media sites, language hurdles can exacerbate problems. Furthermore, digital media translation algorithms routinely give incorrect English translations into various languages [43,44].

In the last year, the number of young people who use digital media has increased considerably. A survey of 1388 students by [45] indicated that 22% of students access digital media sites many times per day, while 60% of students check digital media at least once per day [46,47]. Individual online identities emerge because of digital interaction. The desire to be in touch with friends always and from any location is a powerful drive for utilizing digital media. Digital media may also be used to disseminate information to the general population [48]. Sexting can have unforeseen consequences, such as ruining friendships, relationships, and reputations [49]. Following are some hypotheses based on the foregoing discussion:

**H1.**  *SM is positively related to CH.*

**H2.**  *SM is positively related to CB.*

**H3.**  *SM is positively related to CS.*

**H4.**  *SM is positively related to CE.*

### 2.2. Cyber Engagement (CE)

When a person engages in online communication and sharing of thoughts, images, or language directed at a specific individual via electronic mail or electronic communication, causing that person substantial emotional suffering for no legitimate reason, this is known as cyber engagement [39]. Cyber engagement is described as using email or other kinds of online communication to contact a specific someone for no legitimate purpose, sharing texts, images, or media, and causing that person substantial emotional distress [39]. However, as previously said, cyber participation can directly or indirectly alter the ethical ramifications of activity via behavior intention [50]. Aggressive teens are also more likely to indulge in cyberbullying. The ethical implications of conduct are examined from a variety of perspectives in the research literature. In addition, the study’s findings on the relationship between internet use and ethical conduct are quite constant. According to [10], they discovered that the ethical effects of behavior and cyber engagement beliefs explain a significant portion of intention formation across behavioral domains in adolescents [51], including aggressive acts such as peer cyberstalking, cyberbullying, and cyber harassment and abuse [51,52]. On the other hand, the relationship between internet use and ethical conduct is not well recognized. The following theories were offered based on the previous discussion:

**H5.**  *CE is positively related to CH.*

**H6.**  *CE is positively related to CB.*

**H7.**  *CE is positively related to CS.*

### 2.3. Cyber Bullying (CB)

Cyberbullying is defined as when someone “persistently makes fun of another person online, repeatedly picks on another person through email or text message, or posts anything online about another person that they don’t like” [53]. Cyberbullying, unlike cyberstalking, usually happens between youngsters and is more subtle in nature [53]. Cyberbullying includes text or instant message harassment, password theft, and the use of digital pictures. While some forms of cyberbullying are less harmful since they do not include unlawful activity, others are dangerous and serious, placing victims at grave risk. When attackers imitate a victim by using their profile to participate in illegal activities, such as posting to a pedophile website, this is known as proxy cyberbullying. Angels of Retaliation. The four types of cyberbullying outlined by Kowalski and Limber are Revengeful Nerds (ii), Miserable Girls (iii), and (iv) Careless Cyber Bullies. Cyberbullying and cyberstalking are likely to grow more prevalent as technology advances. Law enforcement officers should be alert, proactive, and inventive in their responses to these offenses. Cyberbullies, unlike traditional bullies, may conceal their identities [54]. Traditional bullying is still more common than internet bullying, according to a meta-analytical study based on 80 past studies [55], which may explain why cyberbullying is considered a sub-category of bullying in general [56].

The following theories were offered based on the previous discussion:

**H8.**  *CB is positively related to CH.*

**H9.**  *CB is positively related to CS.*

**H10.**  *CB is positively related to EE.*

### 2.4. Cyber Harassment (CH)

In the context of cyber harassment, the terms “cyberharassment”, “cyberstalking”, and “cyberbullying” are all used interchangeably. Cyber harassment is defined as a perpetrator’s “desire to intimidate or humiliate the harassment victim” [57]. To characterize online abuse, the terms “cyber harassment”, “cyberstalking”, and “cyberbullying” are all used interchangeably. Cyber harassment is defined by the offender’s “desire to intimidate or humiliate the harassment victim” [57]. According to [58], they describe cyber harassment as any conduct that involves harassing, irritating, scaring, insulting, or threatening someone via email, digital media, or text. In addition, in their research on cyber harassment, Reference [59] investigated the security problems associated with privacy-sensitive information stored on mobile devices, such as smartphones. The harm done to a person by another person using electronic equipment to transmit messages is known as cyber harassment. Cyber harassment might include, for example, creating a Facebook account using another person’s name and looking to harass students. Cyber harassment, as well as cyberbullying and stalking, are the subject of this study. The following theory was presented based on the foregoing discussion:

**H11.**  *CH is positively related to EE.*

### 2.5. Cyber Stalking (CS)

Cyberstalking is the term for stalking on the internet, “an extreme kind of online harassment directed at a single person, causing severe emotional distress while having no legitimate purpose. It is the act of annoyance, alarm, and emotional abuse directed at another person” [60,61]. On the other hand, other students contend that an individual’s right to privacy is “socially constructed”, meaning that it changes through time because of human elements such as culture, legislation, and technology. As a result, cyberstalking is analogous to offline stalking, in which the perpetrators seek to force their victims [62]. Cyberstalking includes sending threatening emails, spamming the victim, and harassing the victim through online interactions [63]. The main distinction between cyber harassment and cyberstalking is the duration of the behavior. To be clear, cyber harassment can only occur once and for a limited period. Internet stalking, on the other hand, can go for weeks, months, or even years [64]. The following theory was presented based on the foregoing discussion:

**H12.**  *CS is positively related to EE.*

### 2.6. Ethical Effect (EE)

Depending on the digital media platform, ethics is a wide term that refers to ethical or unethical acts. Computer ethics is a collection of ethical norms that determines what is considered suitable behavior when using a computer. Computer ethics is a collection of moral rules regulating computer usage in general [65]. As a result, the ethical consequences of students’ activities immediately impact their mental health. “When it comes to using the internet, online ethics entails allowing bad behavior to go unpunished. “We should be honest on the internet and respect the rights and property of others” [65]. Ethics is described as “an issue or scenario that forces a person or organization to choose between decisions that must be regarded as right (ethical) or wrong (unethical)”. The planned behavior model may be used to explore various ethical consequences of behavior problems in relation to digital media and ethical implications of behavior that directly influence students’ psychological health. Because of the flexible characteristics and extensive use of digital media platforms, students have obviously accepted immoral and unexpected behavior.

Decisions concerning one’s conduct must be made daily, posing a variety of ethical and behavioral concerns for digital media users. Across cultures and organizations, digital media now contains a basic way of connection. Around one-third of the world’s population, or 2.2 billion students, use Facebook [66]. Cyber loafing [67,68], cyberbullying [69], cyberstalking [70], unsuitable profile material [71,72,73], identity theft [74], confidentiality [75], and manipulation of users’ information for marketing [76], or staff monitoring are just a few of the ethical issues that have arisen [77,78]. As a result, this research looks at the ethical implications of digital media use, cyber engagement, cyberbullying, cyber harassment, and cyberstalking. It has been suggested that growing moral maturity at a young age is the only method to promote moral accountability since a lack of it might exacerbate ethical ramifications [79].

It is ultimately up to the person to choose whether to act morally or immorally. Given that computer ethics theory is not unified and human ethics tends toward variation, providing such teaching may be difficult [80]. As a result, immoral activity on digital media has a stronger ethical impact than other domains of behavioral conduct. Due to digital and cultural dynamics, customs, and a few other reasons, it may be easier to act unethically on digital media. As a result, information and communication technology (ICT), internet users, and communities are kept in the dark.

Cyberbullying, cyber harassment, and cyberstalking have an impact on students’ ethical conduct. To begin with, cyberbullying, cyber harassment, and cyber stalking have ethical effects on society, including illicit student connections, pornography, stealing, lying, and other ethical issues [81]. Second, cyberbullying, harassment, and stalking all lead to family disintegration [82,83].

Finally, as a result of these crimes, both individuals and society may incur economic and financial costs [59]. In contrast to previous research, which found that students and researchers had a positive attitude toward using digital media for educational purposes and that using digital media can improve academic performance, this study found that students and researchers had a negative attitude toward using digital media for educational purposes [82,84].

## 3. Research Methodology

A total of 1098 persons out of a total of 1169 replied to the poll. However, another 86 people were left out of the research because their responses were incomplete. The replies of 1012 people were imported using the SPSS package software. The participants in this study are undergraduate (Youth) students at a public university who are engaged in digital media. Confirmatory factor analysis is used to test the model’s validity. SmartPLS 3.0 is used to model structural equations with partial least squares (PLS-SEM). The data was collected using a quantitative study framework and questionnaires. The main statistical analysis approach, according to Krejcie and Morgan’s methodology, was PLS-SEM, with SPSS software being used for data analysis [85].

The computed composite reliability was employed to determine an appropriate degree of dependability. Construct validity was assessed in two stages, the first of which was convergent validity and the second of which was discriminant validity. Before examining the hypotheses, the model’s fit appropriateness was confirmed using three procedures: factor loadings, average variance extracted (AVE), and composite reliability. In accordance with [86], discriminant validity was verified using the criterion test, as mentioned in section four.

The structural model was examined in the second step. In the data collection strategy, instruments from the previous study and the main research were used [87,88,89]. The respondents were asked to rate the questionnaire items on a five-point Likert scale, with a ‘5’ indicating strong agreement and a ‘1’ indicating strong disagreement.

The findings may be wrong in some odd cases when the data is not to be used in any research to come up with appropriate conclusions [90].

The questionnaire used in this study was adapted from prior research that was looked at to discover relevant elements to investigate: Cyberstalking (CS) was adapted from [59], cyber harassment (CH) was adapted from [84], cyberbullying (CB) was adapted from [85], cyber engagement (CE) was adapted from [91], and digital media use (SMU) was adapted from [92,93,94].

## 4. Result and Analysis

Gender, age, education level, and specialism were used to categorize demographic characteristics. In terms of gender, 447 (44.2%) of the respondents were male, while 565 (55.8%) were female. 295 (29.2%) of those who responded are between the ages of 18 and 20, while 634 (62.6%) were between the ages of 21 and 24. Furthermore, 56 respondents (5.5%) are between the ages of 25 and 29, and 27 (2.7%) are between the ages of 30 and 34. The respondents’ educational levels are foundation level 147 (14.5%), level one 161 (15.9%), level two 152 (15.0%), level three 404 (39.9%), and level four 148 (14.6%). Finally, 215 (21.2%) respondents are social scientists, 292 (28.9%) respondents are engineers, and 505 (49.9%) respondents are scientific and technology specialists, see Table 1.

A two-step questionnaire data analysis approach was performed, according to [90]. The measuring model’s reliability, convergent, and discriminant validity were investigated initially. The structural model was then evaluated (the indicated links among the constructs and their direction and strength).

### 4.1. Measuring Model Analysis Constructs Reliability

Composite reliability was employed to determine an appropriate degree of reliability, as previously indicated. According to [90], the suggested level for composite dependability is not less than (0.70). Table 2 shows that structures above the 070-cut-off value had a high degree of build dependability, ranging from 0.9125 to 0.9364.

### 4.2. Validity Is Built through Measurement Model Analysis

The degree to which a specific element is manifested in quantifiable components is defined by [90] as construct validity. To ensure construct validity, a systematic assessment of the existing literature was done to find other analysts produced and measured components. Convergent validity can be determined using a variety of methodological methods. For example, factor loadings, AVE, and composite reliability might be used to examine the convergent validity of [90]. In this examination, the suggested values, which range from 0.91 to 0.93, exceeded the acceptable minimum level for composite reliability (0.70). In addition, the acceptable minimum factor loading limit was surpassed.

The findings varied from 0.66 to 0.88 on a scale of one to ten. As indicated in Table 2, the loadings reveal that the factors were allocated to the proper components, which is nearly comparable to 0.50. The factor should be evaluated using the loading of relevant indicators, as stated in [90].

The average variance extracted (AVE) findings varied from 0.59 to 0.72, which is higher than the permissible threshold of 0.5 [90]. The whole confirmatory factor analysis of the statistical model is shown in Table 2. (CFA).

The degree of difference between the items within each construct and the difference between the constructs is assessed to ensure discriminant validity and correlation. At a significance of *p* = 0.001, each construct’s discriminant validity was verified since all AVE findings were substantially above 0.50, as recommended by [95]. According to the discriminant validity measures [90], the square root of the average variance among the relevant items must not be exceeded, as shown in Table 3.

### 4.3. Evaluation of the Model’s Fit

**Figure 2:**
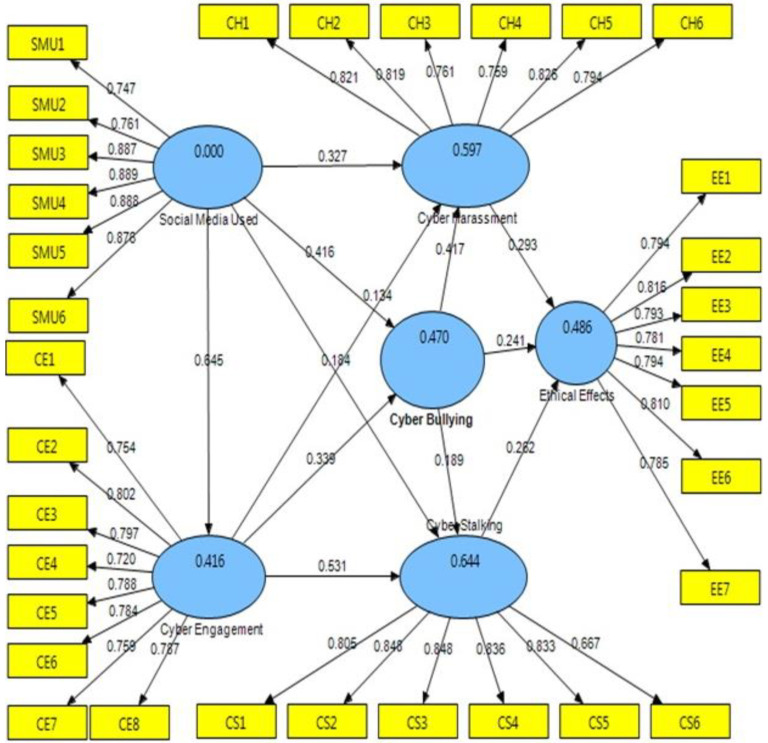
Path coefficients results.

The CMN/DF ratio was 3221, which fell short of the cutoff limit (5.00). GFI (0.967) is an excellent benchmark, CFI (0.944) is exceptional, TLI (0.938) is exceptional, and IFI (0.952) is exceptional. The RMR and RMSEA were both less than the threshold, at 0.31 (0.05) and 0.033 (0.08), respectively. The overall data are shown in Figure 2, which shows that the measurement model suited the structural model well and was adequate for it.

### 4.4. Structural Model Analysis

**Figure 3:**
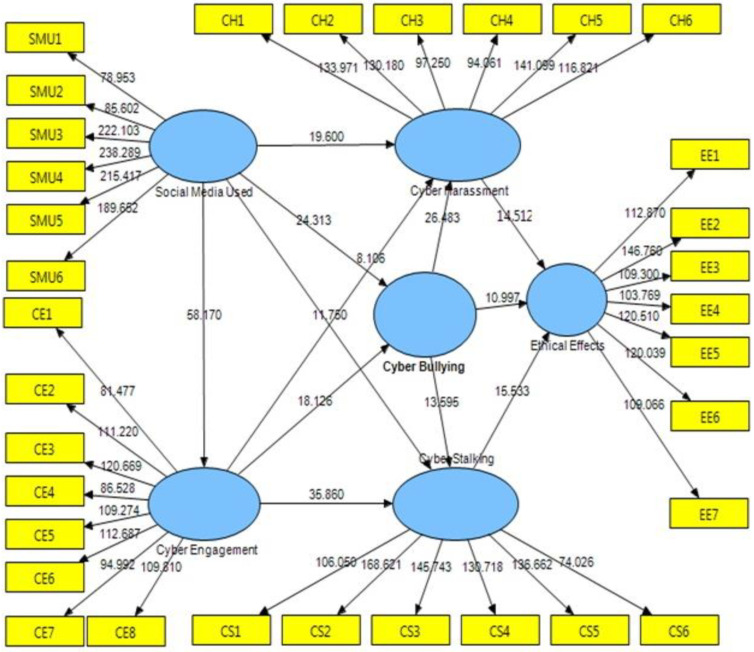
Path coefficients T values.

The Smart PLS 3.0, a PLS technique implementation, was utilized to test the hypotheses of this study and investigate the correlations between the various variables. Figure 1 depicts the identified route coefficients, whereas Figure 2 and Figure 3 depict the hypothesis testing findings. Table 3 also displays the many types of cyberbullying, stalking, harassment, engagement, and digital media in a wide sense, based on ethical implications of behavior that directly impacts students’ psychological health.

The reliability and validity ratings are also included in Table 3. In the following phase of structural equation modeling (SEM), confirmatory factor analysis (CFA) was employed to confirm the hypotheses that had been developed. The results reveal that AVE, CR, and CA values are adequate, demonstrating discriminant validity.

As a result, the data back up all the hypotheses. For the independent variables, digital media use with cyber harassment (0.327), digital media use with cyberbullying (0.416), digital media use with cyberstalking (0.184), and digital media use with cyber engagement (0.184) were used (0.184). (0.645). Cyber harassment (0.134), cyberbullying (0.339), and cyberstalking are forms of cyber engagement (0.531).

Online bullying with cyber harassment (0.417), cyberbullying with cyberstalking (0.189), and cyberbullying with ethical implications on behavior were all discovered to be mediator factors (0.241). Finally, for the dependent variables, cyberstalking with ethical effects behavior that directly harmed students’ psychological health (0.293) and online harassment with ethical effects activity that directly harmed students’ psychological health (0.282). As may be seen in Figure 2, all hypotheses were accepted. Figure 2 and Figure 3 are two instances of this.

The findings support the hypothesis regarding the links between the components and the research model. Table 4 displays the structural framework’s standard errors and the unstandardized coefficient results. As indicated by the solid findings reported in Table 4 for the major statistical measures, the structural framework’s assessment for confirming hypotheses and evaluating the framework’s validity is good.

The first hypothesis, which concerns the relationship between digital media utilization and cyber harassment, realized the following outcomes: β = 0.327228, t = 19.600304, *p* < 0.001. Thus, based on the reported results, the first hypothesis is positive and endorsed. The second hypothesis, the relationship between digital media utilization and cyberbullying, is also positive and endorsed, as the scrutiny pinpoints a positive relationship with digital media (β = 0.415814, t = 24.312815, *p* < 0.001). The third hypothesis, the relationship between digital media utilization and cyberstalking (β = 0.184128, t = 11.750318, *p* < 0.001), is also positive and endorsed. Furthermore, the fourth hypothesis is positive and endorsed, as the scrutiny shows a strong relationship between digital media utilization and cyber engagement (β = 0.644735, t = 58.170153, *p* < 0.001).

The fifth hypothesis, the relationship between cyber engagement and cyber harassment, is also positive and endorsed (β = 0.134230, t = 8.106495, *p* < 0.001). Further, cyber engagement is indicated to be truly and substantially in relation to cyberbullying (β = 0.338938, t = 18.126086, *p* < 0.001). The relationship between cyber engagement and cyberstalking was additionally found to be positive and substantial (β = 0.531131, t = 35.859597, *p* < 0.001). The findings additionally affirm that cyberbullying is greatly correlated with cyber harassment (β = 0.417426, t = 26.482890, *p* < 0.001), concluding that hypothesis number 8 is positive and endorsed. In addition, the findings affirm that cyberbullying is substantially associated with cyberstalking (β = 0.189012, t = 13.595163, *p* < 0.001), thus confirming hypothesis number 9.

Moreover, the results confirm that cyberbullying significantly relates to ethical effects behavior (β = 0.240507, t = 10.997126, *p* < 0.001), and therefore, the hypothesis is supported. The next direct effect is the relationship between cyber harassment and ethical effects behavior that directly affected the psychological health of the students, which was found to be positive and significant as well (β = 0.292765, t = 14.511629, *p* < 0.001). Lastly, findings show that hypothesis 12 is supported where the relationship between cyberstalking and ethical effects behavior that directly affected the students’ psychical health is found to be positive and significant (β = 0.262394, t = 15.532662, *p* < 0.001).

The findings of this study support all of the developed hypotheses, which are consistent with the majority of previous studies that highlighted the impact of digital media use on cyberbullying, cyber harassment, and cyberstalking, which had ethical effects on student behavior that directly affected students’ mental health, e.g., [21,96]. In contrast to other research, such as [49,82,97], which indicated a beneficial influence on student academic performance [98,99,100,101,102], and other studies, such as [49,82]. In terms of hypothesis results, cyberbullying, cyber harassment and cyber stalking are investigated in this study within the confines of a specific digital media network popular among university students.

Despite the idea that digital media is safe, and useful, and promotes digital contacts, the outcomes of this study revealed several negative incidents that were identified and reported. Over half of Facebook users indicated they had been bullied at least once in the previous year. Furthermore, some users have reported objectionable behavior on this site. As the number of young people who use the internet increases, it is expected that they will become more vulnerable to online threats such as data theft and online hacking [21,103]. As a result, traditional protective practices for coping with online hazards are advocated, such as avoiding online strangers and creating online identities [9].

## 5. Discussion and Implications

As a result, younger students are more prone to cyberbullying than older students since they have less experience with cyber interaction, which is less sophisticated and engaging than digital media usage. This is consistent with [19,42]. Cyberbullying, cyber harassment, and cyberstalking pose a major threat to university students, according to the conclusions of this study. As a result, studying the many aspects of cyberbullying, cyber harassment, and cyberstalking in higher education is crucial. University students’ perspectives on online ethical conduct in connection to digital media usage. According to previous research, almost all students believe that digital media is helpful for collaborative digital learning, assignment completion, and general digital interaction with peers [104].

As the internet rose in popularity, so did cyberbullying, harassment, and stalking, which became more common as digital media became more extensively utilized [105]. This corresponds to what we discovered. Because of their capacity to contact one another through the internet, students’ love relationships and friendships are reinforced [106,107]. Despite this, cyberbullying, harassment and stalking have emerged as important internet-related issues. This study’s outcomes show that youngsters who are alone at a computer without their parents peering over their shoulders are more vulnerable to cyberbullying, harassment, and stalking. When monitoring tools are used, the chance of being bullied or harassed by children who use digital media is not considerably reduced, and open communication with parents is crucial.

As a result, and particularly because adult psychological disorders have been linked to mental health issues in children, reducing bullying is crucial [7]. Additionally, cyber harassment can be reduced more effectively if kids are made more aware of the negative repercussions of high-risk internet use, perhaps through outreach initiatives. In addition, unwelcome and harmful communications from strangers may be prevented if guardians’ control and counseling pass on knowledge of proper online behavior. It is feasible to avoid the negative effects of online communication by engaging in appropriate discourse with others and limiting the amount of personal data that is shared. Women and students are especially vulnerable to internet harassment. Furthermore, based on the investigators’ definitions of cyber-bullying, cyber harassment, and cyberstalking, it was common in the existing questionnaire literature for student participants to be asked whether they had victimized someone over the internet or if they had been victimized themselves online [56,91].

They participate in certain unpleasant acts that they do not consider to be cyberbullying, cyber harassment, or cyberstalking, according to their comments. It is worth mentioning that the respondents’ views on cyberbullying, cyber harassment, and cyberstalking have ethical ramifications that directly influence students’ mental health. Furthermore, no significant gender disparities were identified, which is in line with previous research [56,91,108]. Nonetheless, gender discrepancies were discovered to affect the behavior of a specific cyberbullying occurrence. According to one research, females were bullied, harassed, and stalked online more than males. These disparities are also reflected in the offline world. Male respondents were more likely to engage in physical aggression, explicit or direct bullying, and indirect cyber activity. In contrast, female respondents were more likely to engage in direct or indirect cyber activity [109].

Females were also more likely to engage in gossip and other indirect kinds of bullying, harassment, and stalking, as well as be victims of such cyberbullying, harassment, and stalking, whereas males were more likely to engage in violent, threatening cyberbullying. According to current research on cyberbullying, cyber harassment, and cyberstalking [110], our study confirms that females are more frequently the recipients of solicitations to participate in sexual actions via the internet or the receiving of unwanted sexual messages or photos. However, whether girls’ cyber harassment is more widespread and serious than boys’ [111] or whether distinct sorts of harassment are being experienced while pervasiveness and seriousness are equal across genders remains a point of contention [112]. Finally, it is vital to emphasize that females are more prone than males to be bullied, harassed, or stalked.

Despite the fact that [110] found that online bullying and harassment posed a greater risk for poor students, this study found that cyberbullying, cyber harassment, and cyberstalking posed no additional risk to students who did not speak English at home [110]. Students who do not speak English as a first language are more likely than those who do use the internet to accomplish work tasks, according to our research. More studies into the link between cyberbullying, cyber harassment, and cyberstalking are, nevertheless, required. Furthermore, the fact that online bullying, cyber harassment, and cyberstalking were rarely addressed by survey participants was concerning. There appeared to be a variety of reasons why cyberbullying, cyber harassment, and cyberstalking were not reported, including adults’ inability to assist, fear of retaliation, or the compounding of bullying, harassment, and stalking, as well as fears that parents would restrict or take away the mobile phone or online access [9,59].

Finally, the findings of this study back up prior studies [10,54,96,110], suggesting that cyberbullying, cyber harassment, and cyberstalking have ethical ramifications that impact cyber behavior. As a result, web-based communication and relationships should be complex. Therefore, parents and educators should be able to assist young children in developing their digital skills. More study is needed, however, to establish the most effective means of assisting students. It is also important to know the differences between traditional bullying and cyberbullying, cyber harassment and cyberstalking, and the value children place on information and communication technologies. Students should also be encouraged to inform their parents, guardians, instructors, and others about incidents of cyberbullying, harassment, and stalking. This is consistent with previous findings [20,58,113,114].

As a result, we provide some ideas for improving the ethical repercussions of behavior that directly impacts students’ psychological health, as well as for defining and implementing digital media rules and norms. First, students should be given access to digital media privacy settings to reduce unwanted user interactions and data security breaches.

Second, children should be taught how to safeguard their personal data and identities, which would assist in mitigating some of the risks associated with digital media. Third, to avoid problems with internet privacy, more acceptable communication methods, such as phone calls, should be explored among friends. Fourth, by owning a website domain or developing a professional page with links to other relevant pages, students can decrease the internet’s distortion of their personal identity.

Finally, students should be encouraged to use digital media for collaborative digital learning and sharing of knowledge. This is consistent with previous findings [82,104,115]. Therefore, the study’s flaws might open up opportunities for future research by increasing the sample size and concentrating on new industries. They may also investigate the influence of ethical conduct on students’ psychological health in various nations with different cultures, such as cyberbullying, cyber harassment, and cyberstalking.

## 6. Conclusions

In this research on university students, the characteristics that influence ethical conduct that directly affects students’ psychological health through cyberbullying, cyber harassment, and cyberstalking were explored. To conclude, the statistics show that accessing digital media and participating in online activities raises the risk of cyber harassment, bullying, and stalking. These findings highlight the need to consider the roles of digital media and cyber participation in our lives to decrease the negative consequences of cyberbullying, harassment, and stalking.

Finally, there is a pressing need to increase young users’ understanding of how to manage online information to improve the ethical ramifications of conduct that directly impacts students’ psychological health. The current study yielded twelve hypotheses, all of which were confirmed by the data. The hypothesis’ postulated factors were shown to have a meaningful relationship. As a result, future research into cyberbullying, harassment, and cyberstalking should start with this study.

## Figures and Tables

**Table 1:** The demographic factors analysis.

D.F	Categories	Frequency	Percent	D.F	Categories	Frequency	Percent
Gender	Male	447	44.2	Education	Level 1	161	15.9
	Female	565	55.8		Level 2	152	15.0
	Total	1012	100.0		Level 3	404	39.9
Age	18–20	295	29.2		Level 4	148	14.6
	21–24	634	62.6		Total	1012	100.0
	25–29	56	5.5	Specialization	Social Science	215	21.2
	30–34	27	2.7		Engineering	292	28.9
	Total	1012	100.0		Science and Technology	505	49.9
E.D	Foundation	147	14.5		Total	1012	100.0

Note: DF: demographic Factors; E.D: education level.

**Table 2:** Confirmatory Factor Analysis Results.

Factors	Code	Factor Loading	AVE	Composite Reliability	Factors	Code	Factor Loading	AVE	Composite Reliability
Cyber Bullying	CB1	0.8272	0.6828	0.9280	Cyber Stalking	CS1	0.8049	0.7265	0.9299
	CB2	0.8425				CS2	0.8475		
	CB3	0.8407				CS3	0.8477		
	CB4	0.8453				CS4	0.8364		
	CB5	0.8289				CS5	0.8332		
	CB6	0.7711				CS6	0.6667		
Cyber Harassment	CH1	0.8213	0.6351	0.9125	Digital media Used	SMU1	0.7470	0.7116	0.9364
	CH2	0.8187				SMU2	0.7614		
	CH3	0.7607				SMU3	0.8865		
	CH4	0.7586				SMU4	0.8888		
	CH5	0.8256				SMU5	0.8882		
	CH6	0.7944				SMU6	0.8758		
Cyber Engagement	CE1	0.7540	0.5997	0.9229	Ethical behavior online	EE1	0.7935	0.6336	0.9236
	CE2	0.8014				EE2	0.8159		
	CE3	0.7972				EE3	0.7929		
	CE4	0.7199				EE4	0.7806		
	CE5	0.7884				EE5	0.7943		
	CE6	0.7843				EE6	0.8095		
	CE7	0.7592				EE7	0.7846		
	CE8	0.7868							

**Table 3:** Discriminant validity and correlations.

Factors	Code	CB	CH	CS	CE	EE	SMU
Cyber Bullying	CB	0.9148					
Cyber Harassment	CH	0.7052	0.8946				
Cyber Stalking	CS	0.5657	0.5676	0.8573			
Cyber Engagement	CE	0.6068	0.6037	0.7278	0.9435		
Ethical behavior online	EE	0.6121	0.6241	0.5105	0.6366	0.8754	
Digital media Used	SMU	0.6345	0.6794	0.6094	0.6446	0.6005	0.8873

**Table 4:** Hypothesis testing results of structural model.

H	Independent	Relationship	Dependent	Path	S.E.	T. Value	Result
H1	SMU	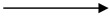	CH	0.327228	0.016695	19.600304	Supported
H2	SMU	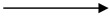	CB	0.415814	0.017103	24.312815	Supported
H3	SMU	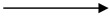	CS	0.184128	0.015670	11.750318	Supported
H4	SMU	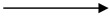	CE	0.644735	0.011084	58.170153	Supported
H5	CE	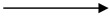	CH	0.134230	0.016558	8.106495	Supported
H6	CE	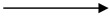	CB	0.338938	0.018699	18.126086	Supported
H7	CE	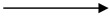	CS	0.531131	0.014811	35.859597	Supported
H8	CB	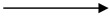	CH	0.417426	0.015762	26.482890	Supported
H9	CB	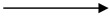	CS	0.189012	0.013903	13.595163	Supported
H10	CB	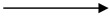	EE	0.240507	0.021870	10.997126	Supported
H11	CH	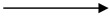	EE	0.292765	0.020175	14.511629	Supported
H12	CS	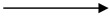	EE	0.262394	0.016893	15.532662	Supported

Note: SE: standard error C.R.: critical ratio or t-value and P: *p*-value.

## Data Availability

Not applicable.

## References

[1] Huang Y.-Y., Chou C. (2010). An analysis of multiple factors of cyberbullying among junior high school students in Taiwan. Comput. Hum. Behav..

[2] Mishna F., Cook C., Saini M., Wu M.-J., MacFadden R. (2011). Interventions to Prevent and Reduce Cyber Abuse of Youth: A Systematic Review. Res. Soc. Work Pract..

[3] Avery E., Lariscy R., Sweetser K. (2010). Social Media and Shared—Or Divergent—Uses? A Coorientation Analysis of Public Relations Practitioners and Journalists. Int. J. Strateg. Commun..

[4] van Laer T. (2014). The Means to Justify the End: Combating Cyber Harassment in Social Media. J. Bus. Ethic.

[5] Sheridan L.P., Grant T. (2007). Is cyberstalking different?. Psychol. Crime Law.

[6] Shi L., Li H., Huang L., Hou Y., Song L. (2022). Does Cyberostracism Reduce Prosocial Behaviors? The Protective Role of Psychological Resilience. Int. J. Environ. Res. Public Health.

[7] Campbell M.A., Slee P., Spears B., Butler D., Kift S. (2013). Do cyberbullies suffer too? Cyberbullies’ perceptions of the harm they cause to others and to their own mental health. Sch. Psychol. Int..

[8] Gabrielli S., Rizzi S., Carbone S., Piras E.M. (2021). School Interventions for Bullying–Cyberbullying Prevention in Adolescents: Insights from the UPRIGHT and CREEP Projects. Int. J. Environ. Res. Public Health.

[9] Lwin M.O., Li B., Ang R.P. (2012). Stop bugging me: An examination of adolescents’ protection behavior against online harassment. J. Adolesc..

[10] Montes Á., Sanmarco J., Novo M., Cea B., Arce R. (2022). Estimating the Psychological Harm Consequence of Bullying Victimization: A Meta-Analytic Review for Forensic Evaluation. Int. J. Environ. Res. Public Health.

[11] Kang K.I., Kang K., Kim C. (2021). Risk factors influencing cyberbullying perpetration among middle school students in Korea: Analysis using the zero-inflated negative binomial regression model. Int. J. Environ. Res. Public. Health.

[12] Starcevic V., Aboujaoude E. (2015). Cyberchondria, cyberbullying, cybersuicide, cybersex: “New” psychopathologies for the 21st century?. World Psychiatry.

[13] Aparisi D., Delgado B., Bo R.M., Martínez-Monteagudo M.C. (2021). Relationship between Cyberbullying, Motivation and Learning Strategies, Academic Performance, and the Ability to Adapt to University. Int. J. Environ. Res. Public Health.

[14] Vaillancourt T., Brittain H.L., McDougall P., Duku E. (2013). Longitudinal Links Between Childhood Peer Victimization, Internalizing and Externalizing Problems, and Academic Functioning: Developmental Cascades. J. Abnorm. Child Psychol..

[15] Hamm M.P., Newton A.S., Chisholm A., Shulhan-Kilroy J., Milne A., Sundar P., Ennis H., Scott S.D., Hartling L. (2015). Prevalence and Effect of Cyberbullying on Children and Young People. JAMA Pediatr..

[16] Bocij P. (2004). Cyberstalking: Harassment in the Internet Age and How to Protect Your Family.

[17] Wei-Jung C. (2020). Cyberstalking and law enforcement. Procedia Comput. Sci..

[18] Tozzo P., Cuman O., Moratto E., Caenazzo L. (2022). Family and Educational Strategies for Cyberbullying Prevention: A Systematic Review. Int. J. Environ. Res. Public Health.

[19] Mitchell K.J., Ybarra M.L., Jones L.M., Espelage D. (2016). What Features Make Online Harassment Incidents Upsetting to Youth?. J. Sch. Violence.

[20] Fenaughty J., Harré N. (2013). Factors associated with distressing electronic harassment and cyberbullying. Comput. Hum. Behav..

[21] Livingstone S., Haddon L., Görzig A. (2011). Risks and Safety on the Internet.

[22] Henson B., Reyns B.W., Fisher B.S. (2013). Fear of Crime Online? Examining the Effect of Risk, Previous Victimization, and Exposure on Fear of Online Interpersonal Victimization. J. Contemp. Crim. Justice.

[23] Gardner D., O’Driscoll M., Cooper-Thomas H.D., Roche M., Bentley T., Catley B., Teo S.T.T., Trenberth L. (2016). Predictors of Workplace Bullying and Cyber-Bullying in New Zealand. Int. J. Environ. Res. Public Health.

[24] Martínez-Monteagudo M.C., Delgado B., García-Fernández J.M., Ruíz-Esteban C. (2020). Cyberbullying in the University Setting. Relationship With Emotional Problems and Adaptation to the University. Front. Psychol..

[25] Arnett J.J. (2008). Adolescencia y Adultez Emergente: Un Enfoque Cultural [Adolescence and Emerging Adulthood: A Cultural Approach].

[26] Ho S.S., Chen L., Ng A.P. (2017). Comparing cyberbullying perpetration on social media between primary and secondary school students. Comput. Educ..

[27] Extremera N., Quintana-Orts C., Merida-Lopez S., Rey L. (2018). Cyberbullying Victimization, Self-Esteem and Suicidal Ideation in Adolescence: Does Emotional Intelligence Play a Buffering Role?. Front. Psychol..

[28] Quintana-Orts C., Rey L., Worthington E.L. (2021). The relationship between forgiveness, bullying, and cyberbullying in adolescence: A systematic review. Trauma Violence Abus..

[29] Faucher C., Jackson M., Cassidy W. (2014). Cyberbullying among University Students: Gendered Experiences, Impacts, and Perspectives. Educ. Res. Int..

[30] Zalaquett C.P., Chatters S.J. (2014). Cyberbullying in college: Frequency, characteristics, and practical implications. Sage Open.

[31] Finn J. (2004). A Survey of Online Harassment at a University Campus. J. Interpers. Violence.

[32] Lindsay M., Krysik J. (2012). Online harassment among college students: A replication incorporating new Internet trends. Inf. Commun. Soc..

[33] Al-Rahmi W.M., Yahaya N., Alamri M.M., Aljarboa N.A., Bin Kamin Y., Moafa F.A. (2018). A Model of Factors Affecting Cyber Bullying Behaviors Among University Students. IEEE Access.

[34] Jantzer A.M., Cashel M.L. (2017). Bullying victimization, college adjustment, and the role of coping. J. Coll. Stud. Dev..

[35] Souza S.B., Simão A.M.V., Ferreira A.I., Ferreira P.C. (2018). University students’ perceptions of campus climate, cyberbullying and cultural issues: Implications for theory and practice. Stud. High. Educ..

[36] Al-Rahmi W.M., Yahaya N., Alamri M.M., Aljarboa N.A., Bin Kamin Y., Bin Saud M.S. (2019). How Cyber Stalking and Cyber Bullying Affect Students’ Open Learning. IEEE Access.

[37] Alkaabi A. (2014). Strategic Framework to Minimise Information Security Risks in the UAE. Ph.D. Thesis.

[38] Mikkola M., Ellonen N., Kaakinen M., Savolainen I., Sirola A., Zych I., Paek H.-J., Oksanen A. (2022). Cyberharassment Victimization on Three Continents: An Integrative Approach. Int. J. Environ. Res. Public Health.

[39] Smith A.M. (2009). Protection of Children Online: Federal and State Laws Addressing Cyberstalking, Cyberharassment, and Cyberbullying. Trends in Internet Research.

[40] Tannenbaum Bullying: How Educators Can Make Schools Safer|Edutopia. https://www.edutopia.org/bullying-making-schools-safer.

[41] Thoma B., Brazil V., Spurr J., Palaganas J., Eppich W., Grant V., Cheng A. (2018). Establishing a Virtual Community of Practice in Simulation: The Value of Social Media. Simul. Health J. Soc. Simul. Health.

[42] Livingstone S. (2008). Taking risky opportunities in youthful content creation: Teenagers’ use of social networking sites for intimacy, privacy and self-expression. New Media Soc..

[43] Ullah N., Mugahed Al-Rahmi W., Alzahrani A.I., Alfarraj O., Alblehai F.M. (2021). Blockchain Technology Adoption in Smart Learning Environments. Sustainability.

[44] Al-Rahmi W.M., Yahaya N., Aldraiweesh A.A., Alamri M.M., Aljarboa N.A., Alturki U., Aljeraiwi A.A. (2019). Integrating Technology Acceptance Model With Innovation Diffusion Theory: An Empirical Investigation on Students’ Intention to Use E-Learning Systems. IEEE Access.

[45] O’Keeffe G.S., Clarke-Pearson K. (2011). The Impact of Social Media on Children, Adolescents, and Families. Pediatric Clinical Practice Guidelines & Policies.

[46] Al-Rahmi A.M., Al-Rahmi W.M., Alturki U., Aldraiweesh A., Almutairy S., Al-Adwan A.S. (2021). Exploring the factors affecting mobile learning for sustainability in higher education. Sustainability.

[47] Al-Rahmi W.M., Yahaya N., Alamri M.M., Alyoussef I.Y., Al-Rahmi A.M., Bin Kamin Y. (2021). Integrating innovation diffusion theory with technology acceptance model: Supporting students’ attitude towards using a massive open online courses (MOOCs) systems. Interact. Learn. Environ..

[48] Al-Rahmi W.M., Alzahrani A.I., Yahaya N., Alalwan N., Kamin Y. (2020). Digital Communication: Information and Communication Technology (ICT) Usage for Education Sustainability. Sustainability.

[49] Al-Rahmi A.M., Shamsuddin A., Alturki U., Aldraiweesh A., Yusof F.M., Al-Rahmi W.M., Aljeraiwi A.A. (2021). The Influence of Information System Success and Technology Acceptance Model on Social Media Factors in Education. Sustainability.

[50] Alalwan N., Al-Rahmi W.M., Alfarraj O., Alzahrani A., Yahaya N., Al-Rahmi A.M. (2019). Integrated Three Theories to Develop a Model of Factors Affecting Students’ Academic Performance in Higher Education. IEEE Access.

[51] Hartshorne R., Ajjan H. (2009). Examining student decisions to adopt Web 2.0 technologies: Theory and empirical tests. J. Comput. High. Educ..

[52] Tan M., Teo T.S.H. (2000). Factors influencing the adoption of Internet banking. J. Assoc. Inf. Syst..

[53] Seo K.K.-J., Tunningley J., Warner Z., Buening J. (2016). An Insight Into Student Perceptions of Cyberbullying. Am. J. Distance Educ..

[54] Choi Y.-J., Shin S.Y., Lee J. (2022). Change in Factors Affecting Cyberbullying of Korean Elementary School Students during the COVID-19 Pandemic. Int. J. Environ. Res. Public Health.

[55] Modecki K.L., Minchin J., Harbaugh A.G., Guerra N.G., Runions K.C. (2014). Bullying Prevalence Across Contexts: A Meta-analysis Measuring Cyber and Traditional Bullying. J. Adolesc. Health.

[56] Mameli C., Menabò L., Brighi A., Menin D., Culbert C., Hamilton J., Scheithauer H., Smith P.K., Völlink T., Willems R.A. (2022). Stay Safe and Strong: Characteristics, Roles and Emotions of Student-Produced Comics Related to Cyberbullying. Int. J. Environ. Res. Public Health.

[57] By S.J. (2008). Cyberharassment: Striking a balance between free speech and privacy. CommLaw. Conspec. J. Commun. Law Policy.

[58] Pereira F., Spitzberg B.H., Matos M. (2016). Cyber-harassment victimization in Portugal: Prevalence, fear and help-seeking among adolescents. Comput. Hum. Behav..

[59] Ojanen T.T., Boonmongkon P., Samakkeekarom R., Samoh N., Cholratana M., Guadamuz T.E. (2015). Connections between online harassment and offline violence among youth in Central Thailand. Child Abus. Negl..

[60] Hitchcock J.A. (2003). Cyberstalking and Law Enforcement. Police Chief.

[61] Parsons-Pollard N., Moriarty L.J. (2009). Cyberstalking: Utilizing What We do Know. Vict. Offenders.

[62] Binsahl H., Chang S., Bosua R. (2015). Identity and belonging: Saudi female international students and their use of social networking sites. Crossings J. Migr. Cult..

[63] Felmlee D., Faris R. (2016). Toxic Ties: Networks of Friendship, Dating, and Cyber Victimization. Soc. Psychol. Q..

[64] Miller C. (2006). Cyber Stalking & Bullying: What Law Enforcement Needs to Know. Annotation.

[65] Moor J.H. (1985). What is computer ethics?. Metaphilosophy.

[66] Appel E. (2014). Cybervetting: Internet Searches for Vetting, Investigations, and Open-Source Intelligence.

[67] Moafa F.A., Ahmad K., Al-Rahmi W.M., Yahaya N., Bin Kamin Y., Alamri M.M. (2018). Develop a Model to Measure the Ethical Effects of Students Through Social Media Use. IEEE Access.

[68] Kwan G.C.E., Skoric M.M. (2013). Facebook bullying: An extension of battles in school. Comput. Hum. Behav..

[69] Huang Y.-Y., Chou C. (2013). Revisiting cyberbullying: Perspectives from Taiwanese teachers. Comput. Educ..

[70] Haron H., Yusof F.B.M. Cyber stalking: The social impact of social networking technology. Proceedings of the ICEMT 2010–2010 International Conference on Education and Management Technology.

[71] Al-Adwan A.S., Albelbisi N.A., Hujran O., Al-Rahmi W.M., Alkhalifah A. (2021). Developing a Holistic Success Model for Sustainable E-Learning: A Structural Equation Modeling Approach. Sustainability.

[72] Al-Maatouk Q., Othman M.S., Aldraiweesh A., Alturki U., Al-Rahmi W.M., Aljeraiwi A.A. (2020). Task-Technology Fit and Technology Acceptance Model Application to Structure and Evaluate the Adoption of Social Media in Academia. IEEE Access.

[73] Peluchette J., Karl K. (2009). Examining Students’ Intended Image on Facebook: “What Were They Thinking?!”. J. Educ. Bus..

[74] Bilge L., Strufe T., Balzarotti D., Kirda E. All your contacts are belong to us: Automated identity theft attacks on social networks. Proceedings of the 18th International World Wide Web Conference.

[75] Alismaiel O.A., Cifuentes-Faura J., Al-Rahmi W.M. (2022). Online Learning, Mobile Learning, and Social Media Technologies: An Empirical Study on Constructivism Theory during the COVID-19 Pandemic. Sustainability.

[76] Plattner M.F. (2011). Comparing the Arab Revolts the Global Context. J. Democr..

[77] Sayaf A.M., Alamri M.M., Alqahtani M.A., Alrahmi W.M. (2022). Factors Influencing University Students’ Adoption of Digital Learning Technology in Teaching and Learning. Sustainability.

[78] Byrnside I. (2007). Six Clicks of Separation: The Legal Ramifications of Employers Using Social Networking Sites to Research Applicants. Vanderbilt J. Entertain. Technol. Law.

[79] Kohlberg L. (1975). The Cognitive-Developmental approach to moral education. Phi Delta Kappan.

[80] Floridi L., Sanders J. (2002). Mapping the foundationalist debate in computer ethics. Ethic Inf. Technol..

[81] Al-Rahmi W.M., Yahaya N., Alturki U., Alrobai A., Aldraiweesh A.A., Omar Alsayed A., Kamin Y.B. (2022). Social media–based collaborative learning: The effect on learning success with the moderating role of cyberstalking and cyberbullying. Interact. Learn. Environ..

[82] Tian B., Hou K.M., Diao X., Shi H., Zhou H., Wang W. (2019). Environment-Adaptive Calibration System for Outdoor Low-Cost Electrochemical Gas Sensors. IEEE Access.

[83] Humpherys S., Drew Hwang U., Poly Pomona Zhongming Ma C., Poly Pomona Ming Wang C., Schwieger D., Kamali A., Carley M., Russell J., Moskal J. (2015). Assessing cyber-bullying in higher education. Inf. Syst. Educ. J..

[84] Huang C.L., Zhang S., Yang S.C. (2020). How students react to different cyberbullying events: Past experience, judgment, perceived seriousness, helping behavior and the effect of online disinhibition. Comput. Hum. Behav..

[85] Krejcie R.V., Morgan D.W. (1970). Determining Sample Size for Research Activities. Educ. Psychol. Meas..

[86] Hair J.F., Sarstedt M., Ringle C.M., Mena J.A. (2012). An assessment of the use of partial least squares structural equation modeling in marketing research. J. Acad. Mark. Sci..

[87] Alqahtani E., Janbi N., Sharaf S., Mehmood R. (2022). Smart Homes and Families to Enable Sustainable Societies: A Data-Driven Approach for Multi-Perspective Parameter Discovery Using BERT Modelling. Sustainability.

[88] Alyoussef I.Y., Al-Rahmi W.M. (2022). Big data analytics adoption via lenses of Technology Acceptance Model: Empirical study of higher education. Entrep. Sustain. Issues.

[89] Haba H.F., Dastane O. (2019). Massive Open Online Courses (MOOCs)—Understanding Online Learners’ Preferences and Experiences. Int. J. Learn. Teach. Educ. Res..

[90] Hair J.F., Ringle C.M., Sarstedt M. (2011). PLS-SEM: Indeed a Silver Bullet. J. Mark. Theory Pract..

[91] Williams K.R., Guerra N.G. (2007). Prevalence and Predictors of Internet Bullying. J. Adolesc. Health.

[92] Alqahtani M.A., Alamri M.M., Sayaf A.M., Al-Rahmi W.M. (2022). Exploring student satisfaction and acceptance of e-learning technologies in Saudi higher education. Front. Psychol..

[93] Baum K., Catalano S., Rand M., Rose K. (2009). Stalking victimization in the United States. The Violence Against Women Act: Elements and Considerations.

[94] Heiman T., Olenik-Shemesh D. (2015). Cyberbullying Experience and Gender Differences Among Adolescents in Different Educational Settings. J. Learn. Disabil..

[95] Bagozzi R.P. (1981). Evaluating Structural Equation Models with Unobservable Variables and Measurement Error: A Comment. J. Mark. Res..

[96] Wolak J., Mitchell K.J., Finkelhor D. (2007). Does Online Harassment Constitute Bullying? An Exploration of Online Harassment by Known Peers and Online-Only Contacts. J. Adolesc. Health.

[97] Yu J., Zo H., Choi M.K., Ciganek A.P. (2013). User acceptance of location-based social networking services: An extended perspective of perceived value. Online Inf. Rev..

[98] Alismaiel O.A., Cifuentes-Faura J., Al-Rahmi W.M. (2022). Social Media Technologies Used for Education: An Empirical Study on TAM Model During the COVID-19 Pandemic. Front. Educ..

[99] Al-Rahmi A.M., Shamsuddin A., Wahab E., Al-Rahmi W.M., Alyoussef I.Y., Crawford J. (2022). Social media use in higher education: Building a structural equation model for student satisfaction and performance. Front. Public Health.

[100] Sayaf A.M., Alamri M.M., Alqahtani M.A., Al-Rahmi W.M. (2021). Information and Communications Technology Used in Higher Education: An Empirical Study on Digital Learning as Sustainability. Sustainability.

[101] Junco R., Heiberger G., Loken E. (2011). The effect of Twitter on college student engagement and grades. J. Comput. Assist. Learn..

[102] Kirschner P.A., Karpinski A.C. (2010). Facebook^®^ and academic performance. Comput. Hum. Behav..

[103] Goudie M., Hutton A., David Hylender C., Niemantsverdriet J., Novak C., Ostertag D., Porter C., Rosen M., Sartin B., Tippett P. (2011). 2011 Data Breach Investigations Report CONTRIBUTORS. Data Breach Investigations Report. https://itb.dk/wp-content/uploads/2020/07/verizon-data-breach-investigations-report-2020.pdf.

[104] Al-Rahmi W.M., Yahaya N., Aldraiweesh A.A., Alturki U., Alamri M.M., Bin Saud M.S., Bin Kamin Y., Aljeraiwi A.A., Alhamed O.A. (2019). Big Data Adoption and Knowledge Management Sharing: An Empirical Investigation on Their Adoption and Sustainability as a Purpose of Education. IEEE Access.

[105] Bannink R., Broeren S., Van De Looij-Jansen P., De Waart F., Raat H. (2014). Cyber and Traditional Bullying Victimization as a Risk Factor for Mental Health Problems and Suicidal Ideation in Adolescents. PLoS ONE.

[106] Valkenburg P.M., Peter J. (2007). Preadolescents’ and adolescents’ online communication and their closeness to friends. Dev. Psychol..

[107] Blais J.J., Craig W.M., Pepler D., Connolly J. (2008). Adolescents Online: The Importance of Internet Activity Choices to Salient Relationships. J. Youth Adolesc..

[108] Law D.M., Shapka J.D., Domene J.F., Gagné M.H. (2012). Are Cyberbullies really bullies? An investigation of reactive and proactive online aggression. Comput. Hum. Behav..

[109] Cullerton-Sen C., Crick N.R. (2005). Understanding the Effects of Physical and Relational Victimization: The Utility of Multiple Perspectives in Predicting Social-Emotional Adjustment. Sch. Psychol. Rev..

[110] Mitchell K.J., Wolak J., Finkelhor D. (2007). Trends in Youth Reports of Sexual Solicitations, Harassment and Unwanted Exposure to Pornography on the Internet. J. Adolesc. Health.

[111] Timmerman G. (2003). Sexual Harassment of Adolescents Perpetrated by Teachers and by Peers: An Exploration of the Dynamics of Power, Culture, and Gender in Secondary Schools. Sex Roles.

[112] Mcmaster L.E., Connolly J., Pepler D., Craig W.M. (2002). Peer to peer sexual harassment in early adolescence: A developmental perspective. Dev. Psychopathol..

[113] Roberto A.J., Eden J., Savage M.W., Ramos-Salazar L., Deiss D.M. (2014). Prevalence and Predictors of Cyberbullying Perpetration by High School Seniors. Commun. Q..

[114] Pettalia J.L., Levin E., Dickinson J. (2013). Cyberbullying: Eliciting harm without consequence. Comput. Hum. Behav..

[115] Andleeb S., Ahmed R., Ahmed Z., Kanwal M. Identification and classification of cybercrimes using text mining technique. Proceedings of the Proceedings-2019 International Conference on Frontiers of Information Technology, FIT 2019.

